# Adaptive optimal allocation of water resources response to future water availability and water demand in the Han River basin, China

**DOI:** 10.1038/s41598-021-86961-1

**Published:** 2021-04-12

**Authors:** Jing Tian, Shenglian Guo, Lele Deng, Jiabo Yin, Zhengke Pan, Shaokun He, Qianxun Li

**Affiliations:** 1 State Key Laboratory of Water Resources and Hydropower Engineering Science, Wuhan University, Wuhan, 430072 China; 2 Changjiang Institute of Survey, Planning, Design and Research, Wuhan, 430010 China

**Keywords:** Hydrology, Hydrology, Climate-change impacts, Projection and prediction, Natural hazards

## Abstract

Global warming and anthropogenic changes can result in the heterogeneity of water availability in the spatiotemporal scale, which will further affect the allocation of water resources. A lot of researches have been devoted to examining the responses of water availability to global warming while neglected future anthropogenic changes. What’s more, only a few studies have investigated the response of optimal allocation of water resources to the projected climate and anthropogenic changes. In this study, a cascade model chain is developed to evaluate the impacts of projected climate change and human activities on optimal allocation of water resources. Firstly, a large set of global climate models (GCMs) associated with the Daily Bias Correction (DBC) method are employed to project future climate scenarios, while the Cellular Automaton–Markov (CA–Markov) model is used to project future Land Use/Cover Change (LUCC) scenarios. Then the runoff simulation is based on the Soil and Water Assessment Tool (SWAT) hydrological model with necessary inputs under the future conditions. Finally, the optimal water resources allocation model is established based on the evaluation of water supply and water demand. The Han River basin in China was selected as a case study. The results show that: (1) the annual runoff indicates an increasing trend in the future in contrast with the base period, while the ascending rate of the basin under RCP 4.5 is 4.47%; (2) a nonlinear relationship has been identified between the optimal allocation of water resources and water availability, while a linear association exists between the former and water demand; (3) increased water supply are needed in the water donor area, the middle and lower reaches should be supplemented with 4.495 billion m^3^ water in 2030. This study provides an example of a management template for guiding the allocation of water resources, and improves understandings of the assessments of water availability and demand at a regional or national scale.

## Introduction

Water resources play a unique role in promoting socio-economic development and protecting the ecological system, which has become a major strategic natural resource of a country^1,2^. However, the uneven spatiotemporal distribution of water resources, the increasing water demand due to the rapid urban expansion and population growth have caused conflicts between social and environment for available water resources^3–5^. The optimal allocation of water resources is one of the most efficient ways to mitigating conflicts, which can effectively allocate the limited available water resources among regions and departments through various engineering measures and non-engineering measures^6^. Furthermore, the identification of the water availability and water demand is the prerequisite for optimal water resources allocation.

However, due to the impacts of climate change and human activities, the heterogeneity in the spatial and temporal distribution of water availability and water demand has intensified, which suggests daunting and urgent challenges on water resources allocation^7–9^. The Intergovernmental Panel on Climate Change (IPCC) report indicated a changing climate in the past that the global average temperature has increased by 1.5 °C within the century from 1906 to 2005. It would accelerate the process of the water cycle^10,11^, and then affect the balance between the water availability and the agricultural water demand in the future. In addition, human activities (e.g., land use/cover change, water transfer projects, water-saving measure, the rapidly development of socioeconomy, etc.) may also alter the distribution and volume of water availability and water demand^1,12–15^. For instance, shifts in farmland area and cover types would result in altered conditions of the underlying surface that was deeply related to the hydrological response^12,14^; the reduced available water volume would be observed in the water donor area because of water transfer project^15^; the application of water saving measure can increase the water-use efficiency and reduce the local water shortage degree^1,13^; the shifts in the regional population and the constitution of different water-use units would significantly change local water demand.

Furthermore, it has been suggested that climate change and human activities will evolve in the future for most basins in the world^16,17^, indicating vital and varying implications for water availability and water demand^18^. Thus, the allocation of water resources that was only based on historical observations (e.g., historical runoff) cannot provide useful references for future water resources planning and management^13^. It is necessary and urgent to study the optimal allocation of water resources based on hydrological cycle simulation under future climate change and future human activities.

A large number of studies have been presented to assess the hydrological response to climate change or anthropic intervention at different scales with the purpose to formulate adaptive water resources planning and allocation strategies for the future^17,19–21^. For example, Chawla and Mujumdar^20^ examined the runoff response in the upper Ganga basin with the VIC model and illustrated that the runoff is sensitive to the change of urban areas and climate change. Pan et al.^21^ studied the hydrological response of 83 catchments in southeastern Australia before and during the meteorological drought period based on the GR4J model, and significant shifts in the catchment water storage capacity have been observed in 62.7% of the meteorological drought period. Yin et al.^17^ studied the temperature scaling of precipitation and storm runoff extremes under different climate conditions over mainland China. However, most of the previous literatures only concentrated on the variations in future water availability due to the projected climate changes, while ignored the impacts of the future anthropic intervention, and did not analyze their impacts on future water resources allocation.

Therefore, the objectives of this study are: (1) to propose a framework to investigate the joint impact of the future climate changes and human activities on water resources allocation; (2) to project future climate by a large set of multi-model ensembles and LUCC scenarios by the CA–Markov model, respectively; (3) to explore the relationship between optimal water resources allocation and water availability (or water demand); (4) to quantify the water supply and compensation measures in the water donor area. The realization of these objectives will make future climate and human activities change projections more reliable, and make the optimal water resources allocation response to future water availability and water demand more adaptive. The remainder of this study consists of several parts: the methodology employed in this study is presented in “Methodology”, followed by the case study and research data in “Study area and data”. Then the results and discussion have been made in “Results and discussion”. Finally, the conclusions are summarized in “Conclusions”.

## Methodology

**Figure 1:**
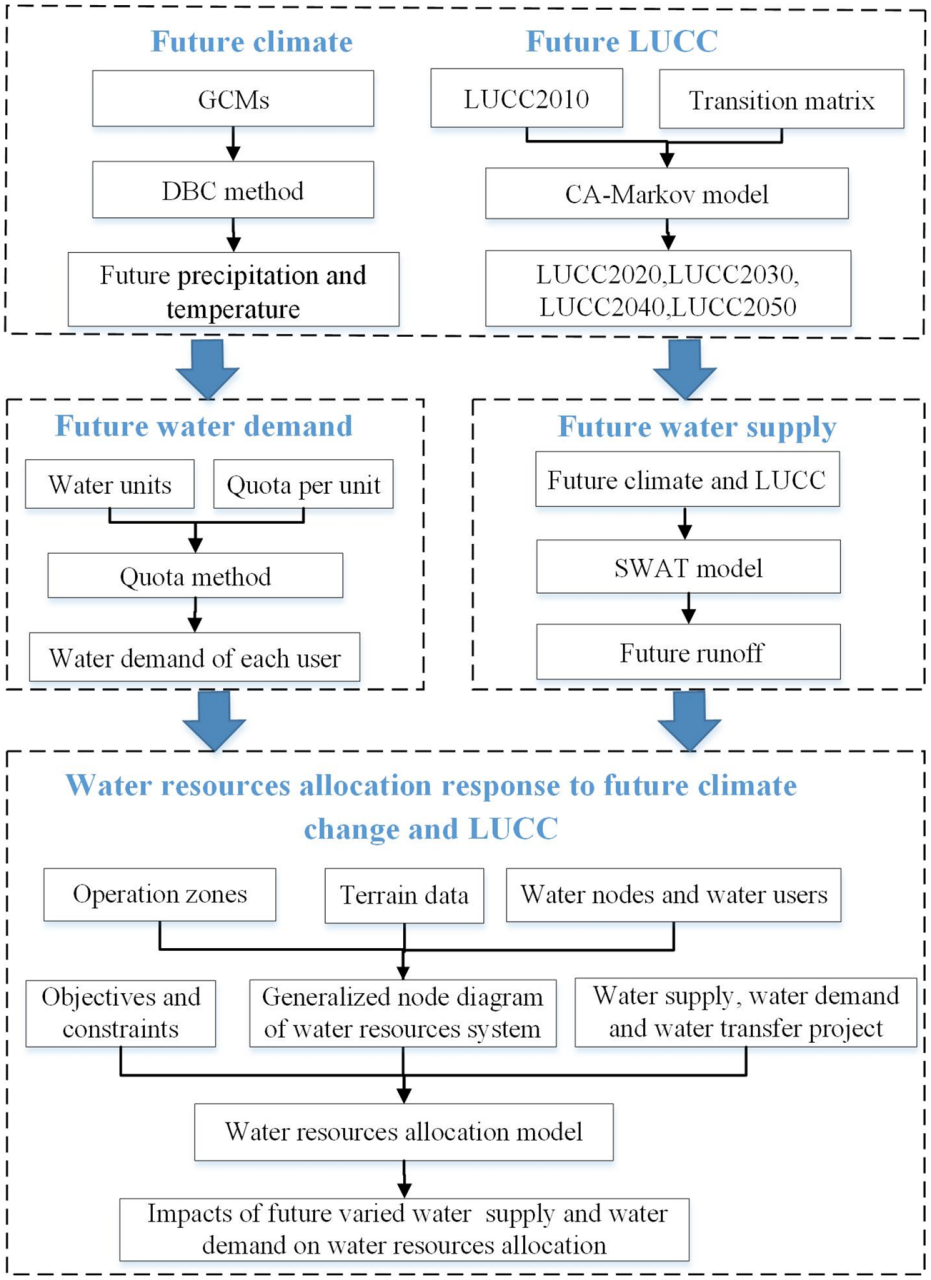
The framework of water resources allocation assessment under future climate change and LUCC. (This figure is generated by Visio2016 software. URL link: https://www.microsoft.com/zh-cn/download/details.aspx?id=55145).

To investigate the effects of future water availability and water demand changes on the optimal allocation of water resources, this assessment scheme is conducted following three procedures: (1) the water availability response to future projected scenarios of climate change and LUCC is studied. A large set of multi-model climate ensembles associated with the DBC method are employed to project future climate scenarios, while the CA–Markov model is used to project future LUCC scenarios. The runoff simulation was based on the SWAT hydrological model under multiple combinations of the projected climate scenarios and LUCC scenarios. (2) the Quota method is used to predict the water demand of various water-using departments under the planning year. (3) the optimal water resources allocation model is established to allocate available water resources on account of the evaluation of the water supply–demand balance structure under different scenarios, and then to quantify the nonlinear impacts of future runoff variation and water demand on water resources planning and allocation. The proposed methodology and procedure are sketched in Fig. 1.

### Future runoff projection

The future temperature and precipitation can be projected by General Circulation Models (GCMs), while the spatial scale of GCM outputs does not match the input scale of the hydrological model in a basin. Therefore, a bias correction method is needed to obtain future temperature and precipitation data in a basin-scale as the inputs of the hydrological model^22^.

In this study, the data of daily precipitation and temperature are generated from ten GCMs of the Coupled Model Inter-comparison Project Phase 5 (CMIP5) under Representative Concentration Pathway 4.5 (RCP4.5). The daily series of precipitation and temperature during 2021–2060 of each GCM were statistically downscaled at twenty-five meteorological stations using the DBC method. The DBC method combined DT (daily translation) and LOCI (local intensity) method^23,24^, assumed that the future and historical climate events have the same deviation in each quantile. When the method is applied to precipitation correction, both the deviation of precipitation and the deviation of precipitation frequency can be considered. Further details are provided by Chen et al.^25^.

Future land-use scenarios of the Han River basin (LUCC2020, LUCC2030, LUCC2040, LUCC2050) were simulated based on the CA–Markov model. The CA–Markov model fully combines the spatial dynamic simulation advantages of the Cellular Automata model and the superiorities of long-term prediction of the Markov model, which can well simulate the change of land use from time and space and is widely discussed and applied^26,27^. Following the general procedures for projecting future LUCC condition, the CA–Markov model are implemented at four stages. (1) The Markov model is used to calculate the transition matrix of LUCC from historical LUCC maps. (2) The simulated transition matrix is modified according to the land use master plan (2006–2020), thus ensuing that the LUCC simulation is not divergent from the actual land use planning. (3) The suitability maps are generated according to the evaluation index in the multi-criteria evaluation module. (4) The spatial distribution of LUCC is simulated by the CA model based on the modified transition matrix and suitability maps.

The SWAT model was selected to simulate runoff, which is a distributed watershed hydrological model developed by the U.S. Department of Agriculture-Agricultural Research Service (USDA-ARS). The hydrological process simulated by SWAT model can be divided into two parts: the land surface part of water cycle (i.e. runoff generation and slope confluence) and the water surface part of water cycle (i.e. river flow routing). The former controls the input of water, sand, nutrients, and chemicals in the main channel of each sub-basin, while the latter determines the transport of water, sand, and other substances from the river network to the outlet of the basin. It can well simulate and predict the changes in hydrological variables on different time scales^28,29^.

In the SWAT model, the whole catchment is firstly segmented into multiple sub-catchments based on the terrain factors and river network distribution. Thus, the hydrological response unit (HRU) is then classified based on the soil type, land use type, and slope area threshold of the basin, and the runoff is calculated separately. Finally, the total runoff of the outlet section is obtained through the river confluence routing^30–33^. According to the water balance principle, the water quantity calculation of the SWAT model follows the following equation.

1 $$SW_{t} = SW_{0} + \sum\limits_{t = 1}^{n} {\left( {R - Q_{s} - ET - S - QR} \right)} ,$$

where $SW_{t}$ represents the final soil moisture content (mm); $SW_{0}$ is the initial soil moisture content (mm); *t* refers to the period of the model simulation; *R*, $Q_{s}$, and *ET* are the precipitation (mm), surface runoff (mm), and actual evapotranspiration (mm), respectively; *S* represents the infiltration and side flow of the bottom layer of the soil profile (mm); *QR* represents the groundwater flow (mm).

The parameters of the SWAT model were calibrated by the SUFI-2 algorithm in SWAT-Calibration and Uncertainty Procedures (SWAT-CUP). The Nash–Sutcliffe coefficient (*NSE*) and the relative error of water volume (*RE*) were used as criteria to evaluate the applicability of the SWAT model in the Han River basin:

2 $$NSE = 1 - \frac{{\sum\nolimits_{t = 1}^{n} {\left( {Q_{o}^{t} - Q_{s}^{t} } \right)^{2} } }}{{\sum\nolimits_{t = 1}^{n} {\left( {Q_{o}^{t} - \overline{{Q_{o} }} } \right)^{2} } }},$$

3 $$RE = \left[ {\left( {\sum\limits_{t = 1}^{n} {Q_{s}^{t} - \sum\limits_{t = 1}^{n} {Q_{o}^{t} } } } \right)/\sum\limits_{t = 1}^{n} {Q_{o}^{t} } } \right] \times 100\% ,$$

where $Q_{o}^{t}$ denotes the observed runoff (m^3^/s); $Q_{s}^{t}$ refers to the simulated runoff (m^3^/s); $\overline{{Q_{o} }}$ is the average of the real runoff observations (m^3^/s); *n* represents the length of observation series.

### Future water demand projection

#### Socioeconomic water demand

The quota method has been proved to be an effective method to project the annual off-stream water demand in the planning year according to Chinese standard (GB/T 51051-2014). The amounts of water demand can be determined by:

4 $$WD_{i,j}^{t} = \frac{{Wq_{i,j}^{t} \times Wa_{i,j}^{t} }}{{UR_{i,j}^{t} }},$$

where $WD_{i,j}^{t}$ refers to the amount of water demand for the *j*th user in the *i*th operational zone at the *t*th period (m^3^); $Wq_{i,j}^{t}$ denotes the water quota unit of water demand user; $Wa_{i,j}^{t}$ represents the water use per activity level; $UR_{i,j}^{t}$ refers to the utilization rate of water user. The water quota units are the amount of water consumption per capita in domesticity user, the amount of water consumption per ten thousand Yuan in the industry user, and the amount of net irrigation water per unit area in the agriculture user, respectively; the water activity level is projected population in the domestic user, projected GDP in the industry user and projected irrigated area in the agriculture user.

#### In-stream ecological water demand

The Tennant method^34^ is used to estimate the in-stream ecological water demand in each area. It is calculated by multiplying the average annual runoff of the operation area by the minimum runoff ratio required in flood season and non-flood season.

5 $$W_{eco,i}^{t} = R_{aa,i} \times \varepsilon_{eco,i} ,$$

where $W_{eco,i}^{t}$ refers to the ecological water demand in the river channel (m^3^); $R_{aa,i}$ represents the mean annual runoff of the *i*th sub-area (m^3^); $\varepsilon_{eco,i}$ denotes the ratio coefficient of the minimum ecological water demand of *i*th sub-area.

### Optimal water resources allocation model

According to the optimal allocation model of water resources as shown below, water resources are allocated among different regions and different water users. The optimal water resources allocation model contains objective function, system constraints, and optimization algorithm.

#### Objective function

The objective function is determined as follows:

6 $$\min f(x) = \sum\limits_{t = 1}^{12} {\sum\limits_{i = 1}^{m} {\sum\limits_{j = 1}^{n} {\alpha_{j} \left( {WD_{i,j}^{t} - x_{i,j}^{t} } \right)} } } ,$$

where *t* denotes to the period, *i* represents the count of sub-regions, and *j* refers the count of water user sectors. *WD*^*t*^_*i,j*_ and *x*^*t*^_*i,j*_ refers to the water demand and water supply of the *j*th sector of the *i*th sub-region, respectively. $\alpha_{j}$ refers to the weight coefficient of water shortage in each water-using sector.

#### System constraints

The system constraints are as follows:

- Water balance constraint of sub-regions
  7 $$W_{{_{i} }}^{t} = \sum\limits_{n = 1}^{{N_{i} }} {\alpha_{n,i} \cdot W_{n}^{t} } + R_{{_{i} }}^{t} + \sum\limits_{k = 1}^{{K_{i} }} {\beta_{k,i} \cdot O_{{_{k} }}^{t} } - \sum\limits_{j = 1}^{m} {(x_{{_{i,j} }}^{t} ){ + }} \sum\limits_{j = 1}^{m} {(cc_{i,j}^{t} \cdot x_{{_{i,j} }}^{t} ) - L_{{_{i} }}^{t} } - TW_{{_{i} }}^{t} ,$$
  where $W_{{_{i} }}^{t}$ is the outlet runoff of the *i*th region at the *t*th period; $W_{n}^{t}$ is the runoff from the *n*th upstream zone, $\alpha_{n,i}$ is the interrelated coefficient between the *n*th and the *i*th region;$R_{{_{i} }}^{t}$ is the sum of the local water volume in the *i*th region; $O_{{_{k} }}^{t}$ is the release from the *k*th reservoir; $\beta_{k,i}$ is the hydraulic connection between the *i*th operational region and the *k*th reservoir; $cc_{i,j}^{t}$ is the return flow coefficient of the *j*th sector in the *i*th region; $L_{{_{i} }}^{t}$ is the quantity of water loss; $TW_{{_{i} }}^{t}$ is the water quantity transferred from of the basin.
- Ecological water requirement of river system
  Water flows in rivers should maintain a healthy environmental condition. That is,
  8 $$W_{i}^{t} \ge EWR_{{_{i} }}^{t} ,$$
  where *EWR*_*i*_^*t*^ is the environment and ecological water requirement of river system in the *i*th operational zone at the *t*th period.
- Water balance equation of reservoir
  9 $$V_{k}^{t + 1} = V_{t}^{k} + I_{t}^{k} - O_{t}^{k} - EV_{t}^{k} ,$$
  where $V_{t}^{k}$ and $V_{k}^{t + 1}$ are the water storage of the *k*th reservoir at the *t*th and (*t* + 1)th period, respectively; $I_{t}^{k}$ is the inflow to the *k*th reservoir at the *t*th period; $O_{t}^{k}$ is the outflow discharge of the *k*th reservoir based on the operation rules. $EV_{t}^{k}$ is the water loss of the *k*th reservoir at the *t*th period.
- Reservoir storage constraint
  10 $$V_{k}^{\min ,t} \le V_{k}^{t} \le V_{k}^{\max ,t} ,$$
  where $V_{k}^{\min ,t}$ and $V_{k}^{\max ,t}$ are the lower and upper bound of the *k*th reservoir at the *t*th period, respectively.
- Water demand constraint
  11 $$x_{{_{i,j} }}^{t} \le WD_{{_{i,j} }}^{t} ,$$
  where $WD_{{_{i,j} }}^{t}$ is the water demand of the *j*th sector in the *i*th operational zone at the *t*th period.
- Water availability constraint
  12 $$\sum\limits_{j = 1}^{l} {x_{{_{i,j} }}^{t} } \le WA_{{_{i} }}^{t} ,$$
  where $WA_{{_{i} }}^{t}$ is the water available in the *i*th operational zone at the *t*th period.
- Non-negativity constraint
  13 $$x_{{_{i,j} }}^{t} \ge 0.$$

#### Optimization algorithm

The genetic algorithm (GA) is originally based on Darwin’s evolution theory^35^, which is an optimization method based on simulating natural gene and natural selection mechanism. According to the rule of “selecting the best and eliminating the inferior”, the algorithm combines the survival of the fittest with the laws of gene variation and reproduction in nature. It adopts random search, selects, crosses and mutates according to the individual's fitness with population as the unit to achieve the optimization purpose^36^.

The optimal allocation of water resources is simulated as a biological evolution problem by GA, the water supply allocated to each water user in each sub-region is taken as the decision variable^37^. By judging the optimization degree of the objective function, the survival of the fittest is carried out, and a new generation of feasible solution set is generated. Therefore, GA is used to solve the water resources allocation model in this study.

## Study area and data

### Study area

**Figure 2:**
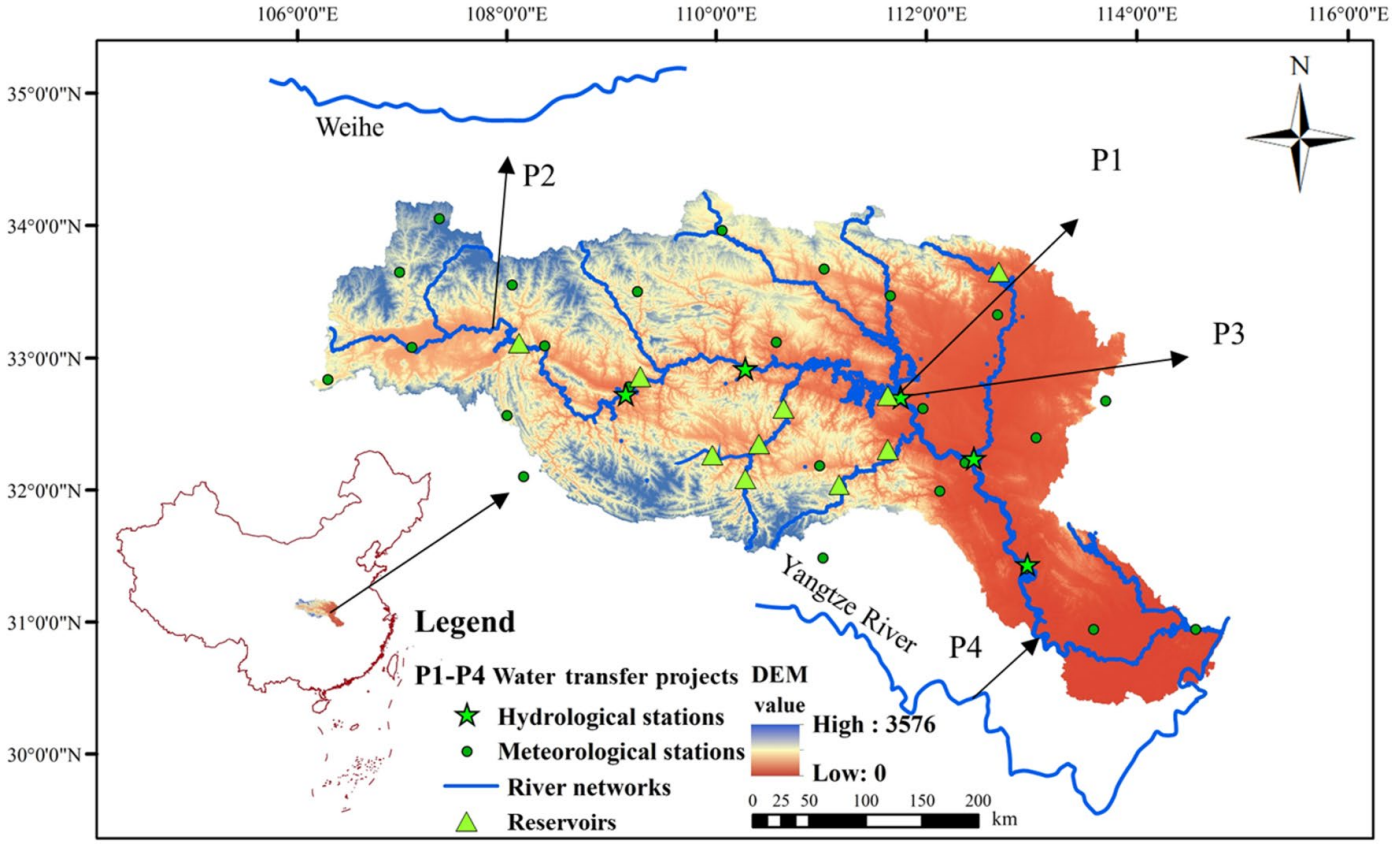
Sketch map of the Han River basin and water transfer projects. (This figure is generated by ArcGIS10.2 software. URL link: http://www.arcgisonline.cn/).

The Han River in China is 1532 km in length, the largest tributary of the Yangtze River. The area of the Han River basin is about 159,000 km^2^, covering 20 prefectures (cities) and 78 counties (cities) in Hubei, Shaanxi, Sichuan, Chongqing, and Gansu provinces. In the basin, the annual average temperature is 12–16 °C, the general trend of annual precipitation is decreasing from southeast and southwest to northwest, the regional variation is between 800 and 1300 mm, and the runoff depth is between 300 and 900 mm. Figure 2. presents the sketch map of the Han River basin, locations of meteorological stations, reservoirs and hydrological stations, and four water transfer projects (detailed information of water transfer projects is shown in Table 1).

**Table 1:** Description of inter-basin and intra-basin water transfer projects.

Project name	Water resource	Water receiving area	Water-use category	Designed annual water transfer amount (billion m^3^)	
				Present	Planning
P1: South-to-North Water Transfer Project	Danjiangkou Reservoir	Henan Province, Hebei Province, Tianjin, and Beijing cities	Domestic, industrial, and agricultural use	9.5	13.13
P2: Han-to-Wei Water Transfer Project	Huangjinxia Reservoir and Sanhekou Reservoirs	Xian, Xianyang, Baoji, Weinan, and Yangling cities	Ecological, domestic, industrial and agricultural users	1	1.5
P3: North Hubei Water Transfer Project	Danjiangkou Reservoir	Xiangyang, Zaoyang, Suizhou, Guangshui and Xiaogan cities	Ecological, domestic, industrial and agricultural users	0.77	0.77
P4: Yangtze-to-Han Water Transfer Project	Yangtze River (Jingjiang River)	Qianjiang, Tianmen, Xiantao cities	Ecological, domestic, industrial and agricultural users	3.1	3.1

The spatial distribution of LUCC types in the basin has large terrain and regional variations, the main types are forest, cropland, grassland, and construction area, the area of water and bare land is very small. The Danjiangkou reservoir services as the boundary of the upper and mid-lower reaches of the Han River basin, provides abundant and clean water to the capital city of China (Beijing) through the middle route of the South-to-North Water Transfer Project. While alleviating the shortage of water resources in the receiving area, the water transfer project also exerts water resources pressure to the water donor area. Hence, the water availability in the Han River basin is not only affected by future climate change and future LUCC, but also by the water transfer projects and the reservoirs’ operation policies. Furthermore, because of the development the socio-economic and urbanization in the basin, the water consumption is increasing and water pollution is becoming intensified serious. Recently, the contradiction of water resources development and utilization in the Han River basin is increasingly significant. Thus, it is urgent to study the optimal allocation of water resources in the Han River basin under the changing environment, which can provide support for the establishment of the strictest water resources management system in the Han River basin.

The construction of inter-basin water transfer projects was one of the most effective approaches to mitigate the unevenness of water resources distribution, which has been used worldwide to solve the water shortage problem of cities. The Han River has served as the water source of several intra-basin and inter-basin water transfer projects, including the middle route of the South-to-North Water Transfer Project, Han-to-Wei Water Transfer Project, North Hubei Water Transfer Project, and Yangtze-to-Han Water Transfer Project. The description of these inter-basin and intra-basin water transfer projects and the designed annual mean water transfer amount is presented in Table 1.

**Figure 3:**
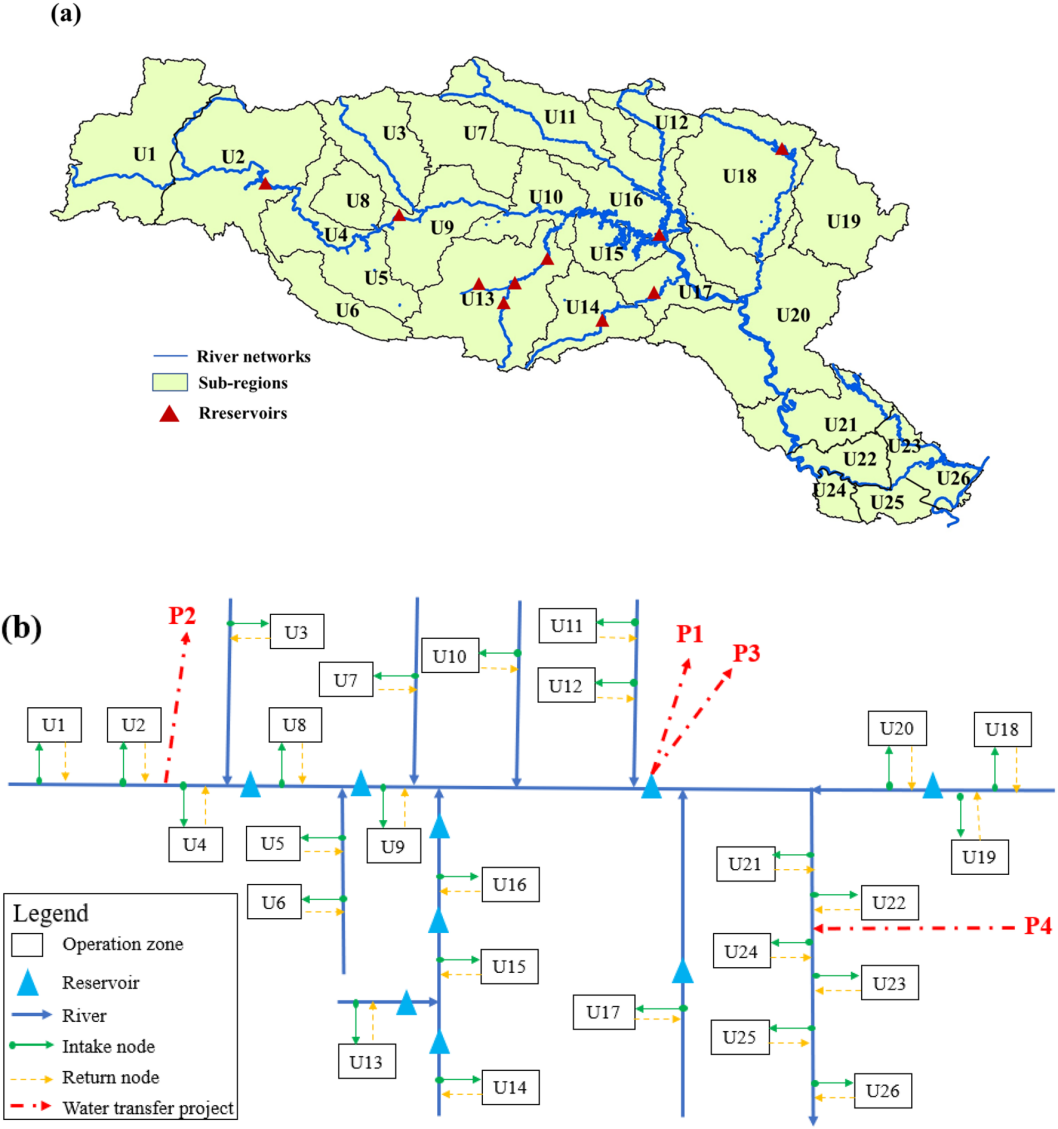
Map of the water supply system in the Han River basin: (**a**) sub-regions in the Han River basin [(**a**) is generated by ArcGIS10.2 software. URL link: http://www.arcgisonline.cn/]; and (**b**) schematic diagram of the Han River basin. [(**b**) is generated by Visio2016 software. URL link: https://www.microsoft.com/zh-cn/download/details.aspx?id=55145].

The water network of the Han River basin is further segmented into 26 sub-regions based on the key nodes, including the water transfer projects, the reservoirs, the sub-regions, the ecological stations and the hydrological gauge stations. The location of each sub-region is presented in Fig. 3a.

To analyze and calculate the supply and demand in the Han River basin, it is necessary to generalize the real water resources system of the study area and draw the node network diagram of the water resources system according to the geographical location and various hydraulic connections between the upper and lower reaches. The node network diagram includes the water node (each calculation unit in the study area), the water source node (including local water source and incoming water), regulation and storage node (large and medium-sized reservoir or large lake with certain regulation capacity), water intake node, water return node, etc. The nodes combined with the water supply route constitute the water resources system network of the whole study area. Taking each sub-region as the water demand control node, according to the hydraulic connection among water system, reservoir, and water users, the node network diagram of the water resources system in the Han River basin is drawn, as shown in Fig. 3b.

### Data

A large set of observational data and model outputs are used, including the data of the Digital Elevation Model (DEM), the data of soil, the data of land use, meteorological inputs, climate series, water demand data, hydrological observations, and reservoirs characteristics. The description and source of each dataset are listed in Table 2.

**Table 2:** Description of the dataset used in this study.

Data type	Description	Data source
DEM data	The spatial resolution of 90 m	Geospatial Data Cloud
Climate data	Daily data from 25 meteorological stations, including precipitation, temperature, wind speed, solar radiation, humidity	China Meteorological Data Service Center (CMDC)
Land use data	LUCC1990, LUCC 2000, LUCC 2010 with a spatial resolution of 1 km	National Earth System Science Data Sharing Infrastructure
Soil data	The spatial resolution of 1 km	Food and Agriculture Organization of the United Nations (FAO)
Hydrological data	Monthly runoff data from 4 hydrological stations	Yangtze River Water Conservancy Commission
Water demand data	Water demand for different users	Integrated Water Resources Planning of Han River basin
Reservoir data	Large or medium-size reservoirs	Hubei Institute of Water Resources Survey and Design

It is informative to note that: (1) the future daily precipitation (Prec), minimum and maximum temperature (T_min_ and T_max_) data are obtained from selected ten GCMs in the CMIP of the Intergovernmental Panel on Climate Change (IPCC). Table 3 presents the basic information of these ten GCMs. The GCMs cover 1961–2005 for the history and 2021–2060 for the future. The GCM data during 1961–2005 is only used to test the applicability of the DBC method. The projected series of the precipitation and temperature of 25 meteorological stations within the study area were corrected using the DBC method under the RCP4.5 emission scenario; (2) referring to the standard of land resources remote sensing survey and classification formulated by the Ministry of Land and Resources of China. The land use in the study area is classified into the following six categories: cropland, forest, grassland, water area, construction area, and bare land. (3) Ankang, Baihe, Danjiangkou, and Huangzhuang hydrological stations alongside the mainstream of Han River are selected to calibrate the parameters of the hydrological model. (4) The development of the socioeconomic communities can affect water demand by changing the water quota units (such as the population, the industrial production, etc.) of various water user sectors. The future demographic has been added in Supplementary Table S2. Moreover, the total industrial productions in the upper, Tangbai river, middle and lower reaches of the Han River basin were 203, 339 and 536 billion yuan in 2016 base year, and are projected to reach 483, 888 and 1250 billion yuan in 2030 planning year, respectively. The data used to calculate water demand are all extracted from the report of Integrated Water Resources Planning of Han River basin compiled by the Changjiang Water Resources Commission.

**Table 3:** The basic information of the selected 10 GCMs.

ID	Model name	Country	Spatial resolution (number of meridional cells × number of latitudinal lattices)
G1	BCC-CSM1.1(m)	BCC, China	128 × 64
G2	BNU-ESM	GCESS, China	128 × 64
G3	CanESM2	CCCMA, Canada	128 × 64
G4	CCSM4	NCAR, USA	288 × 192
G5	CNRM-CM5	CNRM-CERFACS, Canada	256 × 128
G6	CSIRO-Mk3.6.0	CSIRO-QCCCE, Australia	192 × 96
G7	GFDL-ESM2G	NOAA-GFDL, USA	144 × 90
G8	MRI-CGCM3	MRI, Japan	320 × 160
G9	MPI-ESM-LR	MPI-M, Germany	192 × 96
G10	NorESM1-M	NCC, Norway	144 × 96

### Description of scenarios

To assess the response of water resources allocation to future water availability and water demand changes, four scenarios are applied to the study area, which is composed of two water availability scenarios and two water demand scenarios. The impact of water availability change on water resources allocation is assessed based on S1 and S3, (or based on S2 and S4). The influence of water demand change on water resources allocation is investigated by comparing S1 with S2 (or S3 with S4). The detailed information is shown in Table 4.

**Table 4:** The description of the four scenarios.

Scenario	Water availability scenario		Water demand scenario
	Climate scenario	LUCC scenario	
S1	History (1956–2016)	LUCC2010	2016
S2	History (1956–2016)	LUCC2010	2030
S3	RCP4.5 (2021–2060)	LUCC2020–LUCC2050	2016
S4	RCP4.5 (2021–2060)	LUCC2020–LUCC2050	2030

S2–S4 are the future water availability change scenarios (climate change and LUCC simultaneously in the future): RCP4.5 (2021–2030) corresponds LUCC2020; RCP4.5 (2031–2040) corresponds LUCC2030; RCP4.5 (2041–2050) corresponds LUCC2040; RCP4.5 (2051–2060) corresponds LUCC2050.

## Results and discussion

### Projecting future runoff and water resources

#### Performance of the SWAT model

The initial series from 1980 to 2000 is segmented into two periods, i.e., the calibration period (1980–1993) and the validation period (1994–2000). The evaluation indexes of the simulation performances at the calibration and verification periods of the four hydrological stations are presented in Table 5, which shows that the *NSE* value of each hydrological station is greater than 0.8, and the values of *RE* are all within 15%. Due to the large area and complex human activities (e.g. reservoir, water intake, etc.), the natural hydrological region is damaged and the observed data series are not stationary, hence, it is very difficult to simulate the runoff well for all stations (such as the Huangzhuang station during the validation period). Overall, the absolute average values of *NSE* and *RE* during the calibration and validation periods are 0.89, 4.1%, and 0.76, 7.9%, respectively, indicating that the SWAT model performs well in the study area.

**Table 5:** Calibration and validation results of the SWAT model.

Number	Stations	calibration period (1980–1993)		validation period (1994–2000)	
		*NSE*	*RE*/%	*NSE*	*RE*/%
1	Ankang	0.93	2.4	0.83	8.1
2	Baihe	0.93	− 0.3	0.78	− 1.9
3	Danjiangkou	0.87	12.1	0.75	14.5
4	Huangzhuang	0.82	− 1.4	0.66	7.1
Average of the absolute value		0.89	4.1	0.76	7.9

#### Water availability under future climate change and LUCC

**Figure 4:**
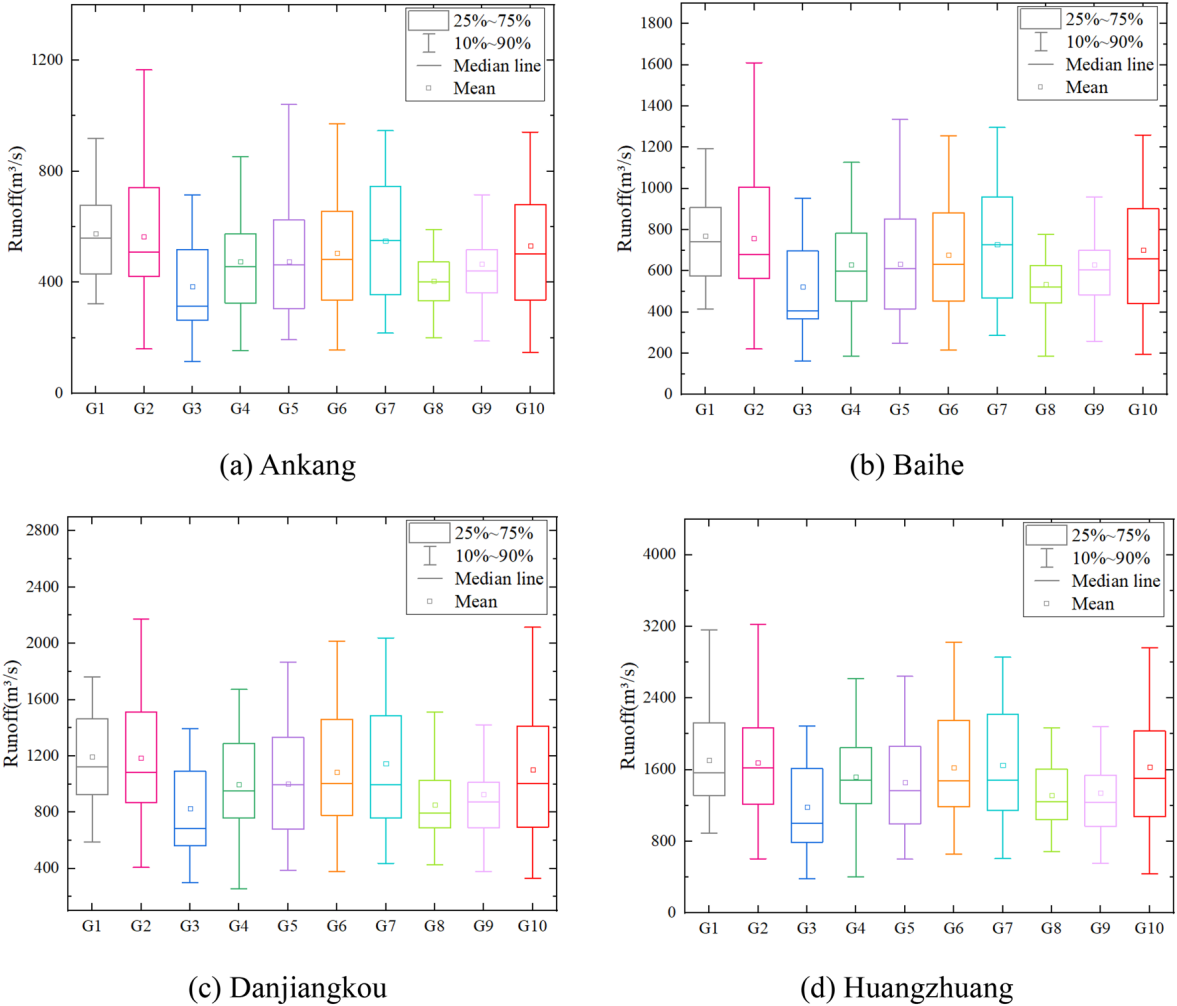
Comparison of future runoff series at various hydrological stations simulated by ten GCMs.

The daily precipitation and temperature in the future (2021–2060) after bias correction are generated by ten GCMs are then used to force the SWAT model for runoff simulation, the statistical characteristics of simulated runoff series by ten GCMs at the mainstream stations of the Han River basin are compared in Fig. 4. From the figure, we can see that, the simulation effects of GCM on the extreme value and mean value are not the same. Some GCM simulation series have great interannual variation (such as G2, G7), while some GCM simulation results have little interannual variation (such as G8, G9). The results show that the runoff simulation error is caused by GCM precipitation error input into the hydrological model, which is consistent with the previous research results^38^. Therefore, to reduce the uncertainty of the single climate model for runoff simulation, this study uses the multi-GCMs average results as the input of the hydrologic model to predict future runoff and water resources.

The changes in annual average local water availability from ten GCMs in the Han River basin under future climate change and future human activities conditions are listed in Table 6. It should be noted that the runoff value in the historical period is simulated from the SWAT model. The results show that: the future water availability in all areas will increase in the future compared to the base period (1956–2016). Under the RCP4.5 concentration pathway, the water availability change rate of the upper, Tangbai river, and middle and lower reaches in the Han River basin are + 5.26%, + 2.82%, and + 2.81%, respectively. From the perspective of water resources distribution in a year, the local water resources in the flood (non-flood) season under future RCP4.5 scenario are projected to increase (decline). The water availability changing rates of the upper, Tangbai river, middle and lower reach reaches in the flood season are + 19.57%, + 3.05%, and + 10.61%, while the rates in the non-flood season are − 15.88%, − 0.82%, and − 1.25%, respectively. The results show that the uneven distribution of local water resources may be more severe in the future. Overall, the change rate of local water availability in the Han River basin is + 4.47% under the RCP4.5 concentration pathway. The impact of precipitation change on runoff is direct, and the relationship between them is positive, while the impact of temperature change on runoff is indirect with a negative correlation.

**Table 6:** Future local water availability change compared with the historical period of the Han River basin (billion m^3^).

Area	Flood season			Non-flood season			Annual		
	Historical	RCP4.5	Change rate	Historical	RCP4.5	Change rate	Historical	RCP4.5	Change rate
The upper reach	46.88	56.06	19.57%	14.58	12.27	− 15.88%	36.88	38.82	5.26%
The Tangbai river reach	7.04	7.25	3.05%	1.58	1.57	− 0.82%	5.17	5.32	2.82%
The middle and lower reaches	15.15	16.69	10.16%	5.62	5.55	− 1.25%	12.46	12.81	2.81%
Sum	69.07	79.80	15.54%	21.78	19.56	− 10.21%	54.51	56.94	4.47%

**Figure 5:**
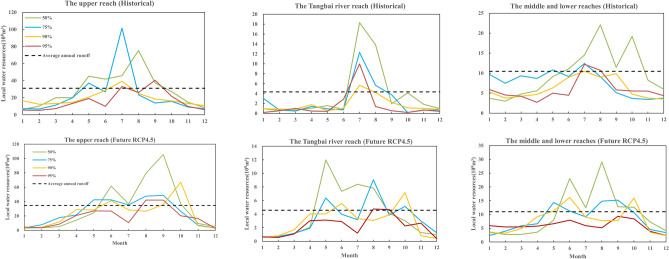
Local water resources under different inflow frequencies in history and future.

In the multi-year average runoff series, the local water resources under four water inflow frequencies (50%, 75%, 90%, and 95%) in the history and future (RCP4.5) are selected for analysis, and the results are shown in Fig. 5. No matter in the upper, Tangbai river, or the middle and lower reaches, the average local water resources of the four inflow frequencies under future RCP4.5 scenario is greater than the historical inflow conditions. At the same water flow frequency, the local water resources in the upper reach are the largest, followed by the middle and lower reaches, and finally in the Tangbai river reach.

### Water demand in the planning year

**Figure 6:**
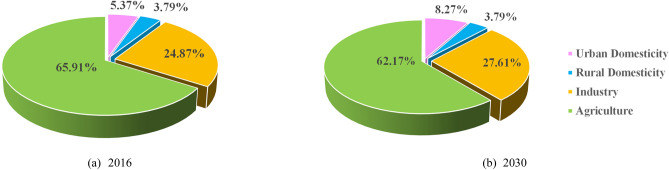
Proportion of different water user sectors to total off-stream water demand (75%).

Based on the water demand prediction model, the water demands in the five water users in 2016 base year and 2030 planning year are estimated. The in-stream water demand is determined by the Tennant method based on the local water resources. The annual water demand of different water user sectors is listed in Table 7. Taking the water demand scheme under the water flow frequency of 75% as an example, the proportion of different water user sectors to total off-stream water demand is shown in Fig. 6.

**Table 7:** Water demand projection of the Han River basin in 2016 base year and 2030 planning year (billion m^3^).

Year	Area	Domesticity		Industry	Agriculture				Ecological	Sum			
		Urban	Rural		50%	75%	90%	95%		50%	75%	90%	95%
2016	The upper reach	0.26	0.20	0.59	2.66	2.84	3.10	3.12	10.34	14.06	14.23	14.50	14.52
	The Tangbai river reach	0.22	0.18	0.72	2.84	3.36	3.86	3.89	1.41	5.36	5.89	6.38	6.41
	The middle and lower reaches	0.54	0.34	3.41	5.56	6.31	7.07	7.58	3.52	13.38	14.13	14.89	15.40
	Sum	1.02	0.72	4.72	11.06	12.51	14.03	14.59	15.27	32.80	34.25	35.77	36.33
2030	The upper reach	0.47	0.19	0.71	2.59	2.76	3.00	3.01	11.48	15.44	15.61	15.85	15.86
	The Tangbai river reach	0.35	0.17	0.84	2.77	3.32	3.78	3.83	1.84	5.97	6.52	6.98	7.03
	The middle and lower reaches	0.76	0.35	3.69	5.16	5.72	6.41	6.83	3.81	13.77	14.33	15.02	15.44
	Sum	1.57	0.72	5.24	10.52	11.8	13.18	13.67	17.13	35.18	36.46	37.84	38.33

According to Table 7, under the water flow frequency of 50%, 75%, 90%, and 95% in 2016, the total water demand of the Han River basin is 32.80, 34.25, 35.77 and 36.33 billion m^3^, respectively. In 2030 planning year, the total water demand of the Han River basin is 35.18, 36.46, 37.84 and 38.33 billion m^3^, respectively. The results show that the total water demand increases with the increase in water flow frequency, and there will be a slight increase in 2030 compared with those in 2016. From the perspective of different areas in 2030 planning year, we can see that the total water demand in the upper reach is the largest, which is 15.44, 15.61, 15.85 and 15.86 billion m^3^ under the water flow frequency of 50%, 75%, 90% and 95%, respectively, accounting for 43.89%, 42.81%, 41.89% and 41.38% of the total water demand in the Han River basin. Followed by the middle and lower reaches with 13.77, 14.33, 15.02 and 15.44 billion m^3^ under the water flow frequency of 50%, 75%, 90%, and 95%, respectively. The water demand in the Tangbai river reach is the least.

From the perspective of different water users, with the increasing population and the improvement of living standards, the demands in domestic water users show a gradual increasing tendency, among which the urban domestic water demand increases compared with the slight reduction in rural domestic water demand. Although the industrial water consumption quota in 2030 planning year is projected to decline in reference with 2016 base year, the total industrial water consumption will increase rapidly due to industrialization, while the agricultural water demand will drop due to the popularization of water-saving technologies. In the upper, Tangbai river, middle and lower reach reaches, the total industrial water demand will increase by 0.12, 0.12 and 0.28 billion m^3^, while the agricultural water demand will decrease by 0.08, 0.04 and 0.59 billion m^3^, respectively.

### Adaptive optimal allocation of water resources

**Figure 7:**
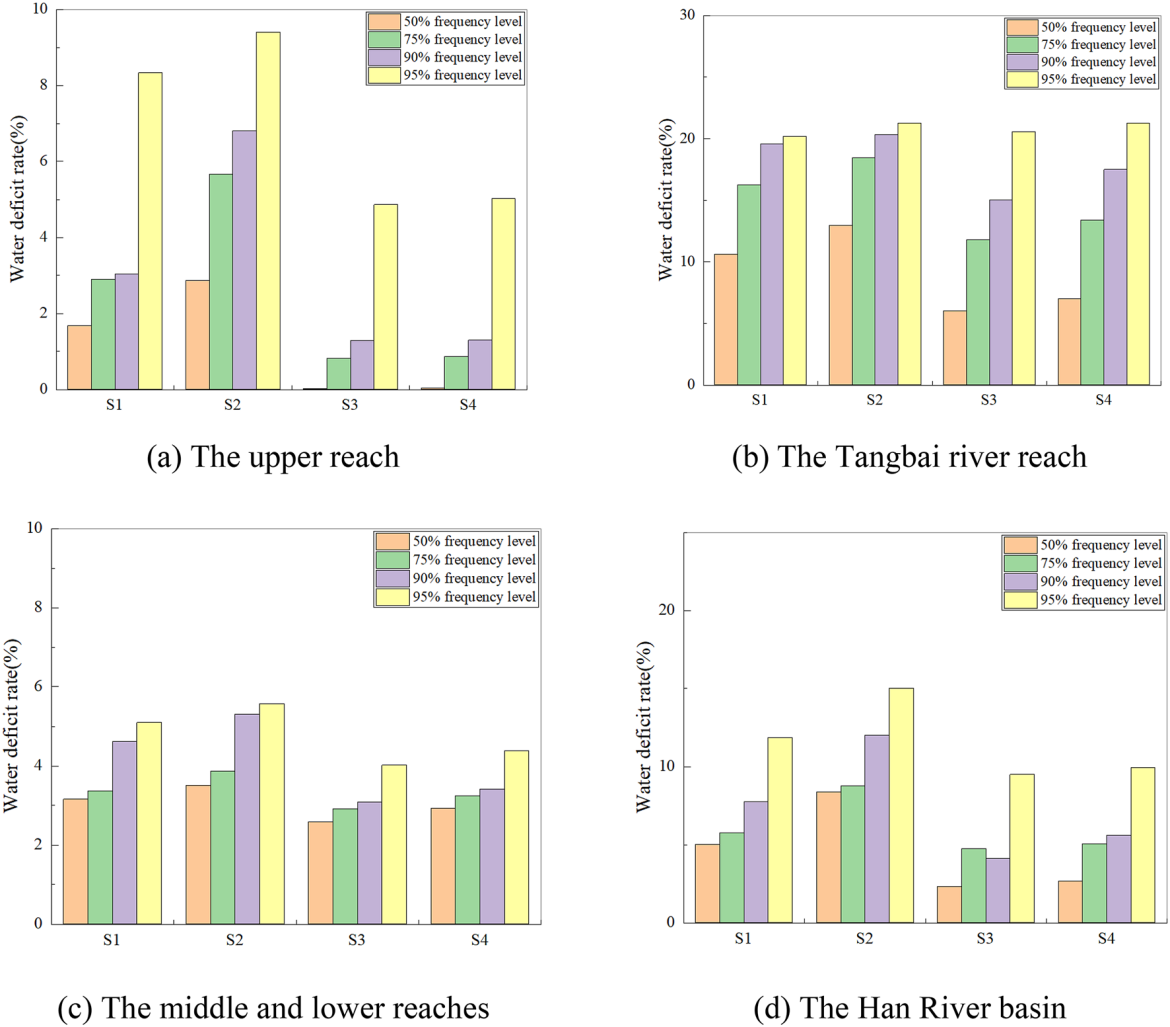
Water shortage rate in different water users under different scenarios.

Based on the water availability and water demand prediction results of the Han River basin under future climate scenarios, future LUCC scenarios and water transfer projects, the water supply of different water users in sub-regions of the case study is calculated according to the established water resources allocation model. The water deficit rate of different water users under the four scenarios is shown in Fig. 7.

**Figure 8:**
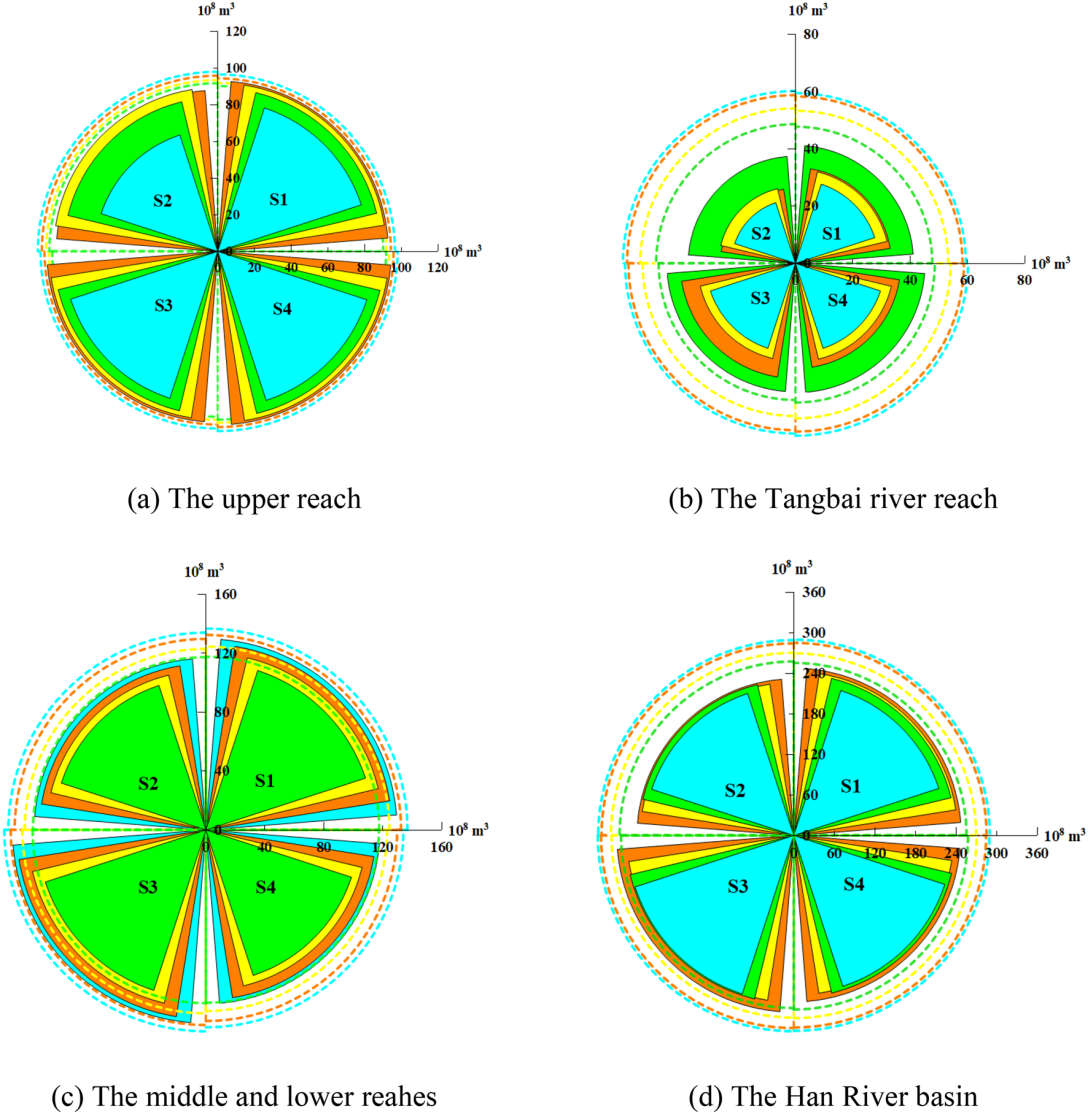
Diagram of water supply and demand under different scenarios in each area, on which  and  represent the amount of allocated water and water demand at 50%, 75%, 90% and 95% frequency level, respectively. (This figure is generated by Origin2020 software. URL link: https://www.originlab.com/2020).

Changes in future water availability are caused by future climate change and LUCC. Figure 8 shows the assessment results of future climate change and future LUCC on the water resources allocation in the Han River basin. In Fig. 8, the color of the arc line and arc polygons mean the water flow frequency level of available water resources (i.e. the probability of that drought event will not occur), while the value of arc line and arc polygon represent the water demand and the water allocation result. The interval of the arc lines and the arc polygons is the water deficit value. Four scenarios described in “Description of scenarios” are shown in the four quadrants of the Cartesian coordinate system. What’s more, there are four water flow frequencies (50%, 75%, 90%, and 95%,) used for analysis under each scenario. Hence, there are 16 (2 × 2 × 4) kinds of water resources allocation results of each sub-region to show the effects of changing environment on optimal allocation of water resources.

As shown in Figs. 7 and 8, the water shortage rate of the Han River basin is not large under the condition of historical inflow. As for the results under S1, the total water deficit ratio in the Han River basin is 5.02%, 5.74%, 7.76%, and 11.87%, at the four frequencies 50%, 75%, 90%, and 95%. From the spatial distribution of water shortage rate, the total water deficit ratio in the upper reach is 1.68%, 2.89%, 3.03% and 8.33% in the Tangbai river reach is 10.63%, 16.27%, 19.57% and 20.20%, and 3.16%, 3.37%, 4.62%, 5.10% in the middle and lower reaches at the four frequencies 50%, 75%, 90% and 95%. The results show that: at the same level year, the total water shortage ratio increases slowly along with fewer water resources. The water shortage ratio in the Tangbai river reach is much higher than that in the upper reach, and middle and lower reaches, which is since the local water resources in the Tangbai river reach are the least, and there is a large gap due to the lack of effective regulation, because of the large water demand and the limitation of reservoir function in the Tangbai river reach. When comparing the supply and demand balance results of the two years, it can be seen that under the same water flow frequency, the total water deficit ratio shows an increasing trend with the promotion of 2016 base year range to the planning level year 2030. Under the same water demand scheme, the water deficit in the future is slightly smaller than that in the historical period.

#### Water resources allocation response to future water availability change

The impact of water availability change on water resources allocation is assessed based on S1 and S3 (or based on S2 and S4) according to the same water demand scenario. Figure 7 shows the water shortage rate in different areas and the whole basin under four scenarios. We can see that: (1) judging from the water shortage rates in different scenarios, when comparing the water deficit rates of S1 with S3, or S2 with S4, we can find that the water shortage rate is sorted as S1 > S3, S2 > S4, respectively. It seems to contradict the result of water availability under the three scenarios (the water availability is S3 > S1, S4 > S2). (2) judging from the water shortage in different water areas in any scenario, the water shortage rate in the Tangbai river reach is the highest, which is 12.06% 21.19%, 7.21%, 8.45% from S1–S4 under the water flow frequency of 50%. Followed by the upper reach, and finally is the middle and lower reaches. This is related to that the number of reservoirs in the Tangbai river reach is small and the regulation capacity of reservoirs is limited. What’s more, the major of the water demand in the Tangbai river reach concentrates on agriculture, and the process of inflow could not match the process of water demand fitly. In terms of spatial distribution (shown in Supplementary Fig. S2), the mainstream of Han River flows through the upper, middle and lower reach reaches. The Tangbai river is a tributary of Han river which is unable to extract water from the mainstream. Combined with the above reasons, the water shortage rate in the Tangbai river reach is the highest. Overall, the effects of future climate change and LUCC will decrease water shortage in the Han River basin.

From the results of “Water availability under future climate change and LUCC”, the average water availability change rate in the upper reach, Tangbai river reach, middle and lower reaches of the Han River basin under the RCP4.5 concentration pathway are + 5.26%, + 2.82%, and + 2.81%, respectively, when compared with the historical water availability. However, taking the water demand scheme in 2016 base year as an example, the water shortage rate (S3–S1) will change − 1.66% in the upper reach, − 4.61% in the Tangbai river reach, and − 0.57% in the middle and lower reaches at the water flow frequency of 50% under the RCP4.5 concentration pathway, when compared with the historical water shortage rate. The results show that a nonlinear relationship has been identified between the optimal water resources allocation and the water availability, which may be explained by the reason that the water transfer projects can change the distribution of water supply on the spatial scale, and the reservoirs can change the water supply on the time scale. Therefore, the impacts of future climate change and human activities on water availability does not equal to the impacts on optimal water resources allocation.

#### Water resources allocation response to future water demand change

The results of an impact assessment of water demand change on water resources allocation are investigated by comparing S1 with S2 (or S3 with S4) according to the same water availability scenario. As shown in Fig. 7, in the same case of water availability, the water deficit rates of all areas are increasing under the water demand scenario of 2030 compared with the water demand scenario of 2016 (S2 > S1, S4 > S3), which is caused by the increasing water demand shown in Table 7. As for the different areas, Fig. 8 shows the relationship between water demand and water supply. And it can be found that at the upper reach of the Han River basin, water supply is almost equal to water demand regardless of any scenario except for at the water flow frequency of 95%, which shows that the increased water availability can meet the increased water demand. As for the Tangbai river reach of the Han River basin, the effects of future water demand change will increase water scarcity in the case of insufficient water availability.

From the results of “Water demand in the planning year”, taking the water flow frequency of 50% as an example, the total off-stream water demand in 2030 will change + 2.97% compared with the historical water demand. Then the total water shortage rate of the basin will change + 4.62% (S2–S1) and + 3.49% (S4–S3) when compared with the historical water shortage rate. This result shows that a linear relationship has been identified between the optimal allocation of water resources and the water demand. That is the increase in water demand will cause an increase in water shortage under the condition of constant incoming water, which will aggravate the contradiction between supply and demand.

In general, in the same planning year, the total water deficit slowly increases along with fewer water resources. Under the same water demand scheme, the water deficit in the future is slightly smaller than that in the historical period. The results show that under the situation where water availability and water demand varied simultaneously in the future, the response result of water resource allocation is that water shortage in the basin will decrease.

#### Analysis of water replenishment

According to the working conditions considered in years 2016 and 2030 (Table 1) and the dispatching regulations of Danjiangkou reservoir, the actual water quantity of each water transfer project and actual discharge from Danjiangkou in 2016 and 2030 are obtained except for the water resources allocation results for each water sectors of sub-regions. Results under S1 and S2 are used for analysis, the annual average water shortage of each water transfer project and the in-stream water ecology are shown in Table 8. It can be seen that when the natural inflow runoff of Danjiangkou adopts the 1956–2016 series, the average annual water deficit of P1, P2, P3, and in-stream ecology of the mainstream of Han River in 2016 base year is 1.40, 0, 0.80, and 0.10 billion m^3^, respectively. In the 2030 planning level year, the average annual water deficit of P1, P2, P3, and the in-stream ecology in the mainstream of Han River is 3.09, 0, 1.30, and 0.11 billion m^3^, respectively. It should be noted that the Han-to-Wei water transfer project (P2) is directly deducted from the inflow of the Danjiangkou reservoir, and there is no water shortage of P2.

**Table 8:** The annual average water shortage (billion m^3^).

Year	P1	P2	P3	In-stream ecology	Sum
2016	1.40	0.00	0.80	0.10	2.30
2030	3.09	0.00	1.30	0.11	4.50

**Figure 9:**
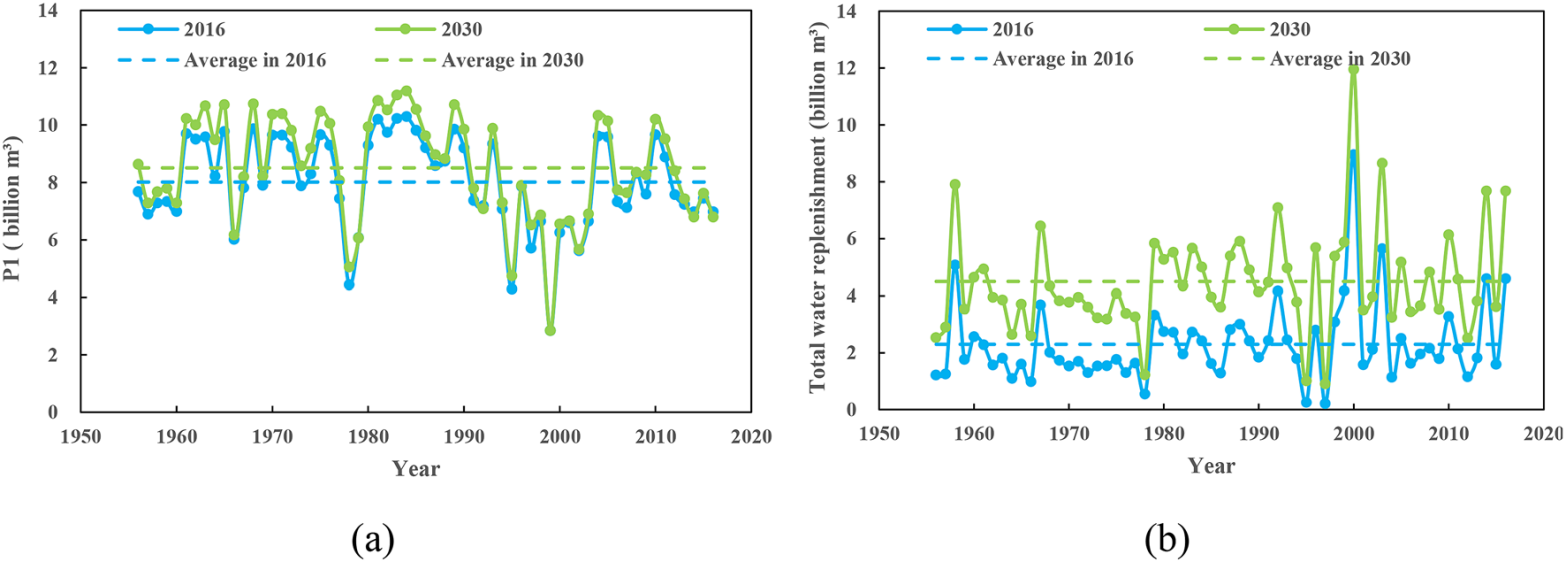
(**a**) Actual water transfer quantity of South-to-North Water Transfer Project (P1) obtained from the water resources allocation model; (**b**) Actual water replenishment required in the middle and lower reaches of Han River basin obtained from the water resources allocation model.

According to the water resources allocation results, the average annual volume of South to North Water Transfer project in 2016 is 8.011 billion m^3^, and that in 2030 is 8.505 billion m^3^. The actual water transfer quantity process of the South-to-North Water Transfer Project (P1) under the two water demand schemes are shown in Fig. 9a. As can be seen from the figure, except for the difference in water quantity, the water diversion process trend under the two-level years is almost identical, which is since the trend of water inflow from Danjiangkou and the water supply sequence of Danjiangkou reservoir for each working condition are completely consistent under the two scenarios. Figure 9b shows the amount of water diverted from the river for each year from 1956 to 2016. Comparing the water replenishment process of the two water demand schemes, it can be seen that the change trends are completely the same. The difference is: in 2016 base year, the annual average water replenishment is 2.301 billion m^3^, the annual average water replenishment is 4.495 billion m^3^ in 2030 planning year.

### Uncertainties discussion

**Figure 10:**
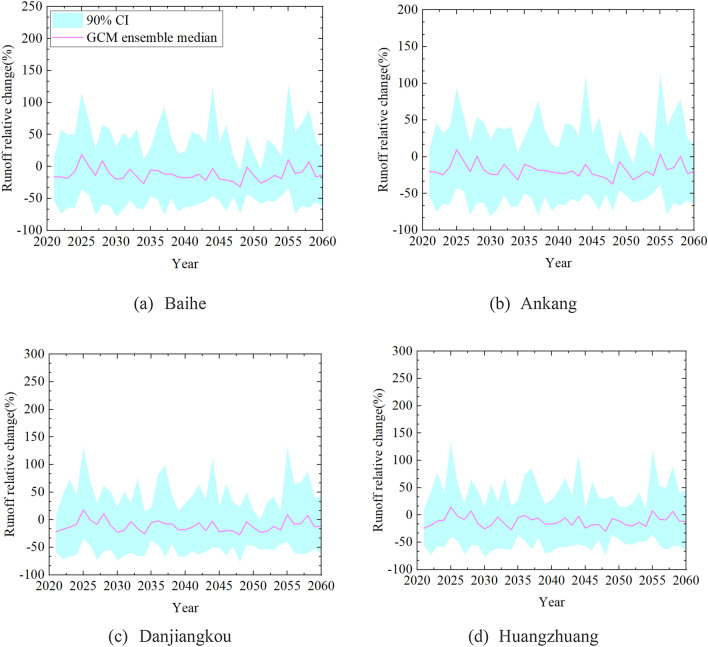
Uncertainty range of future runoff projection at four hydrological stations in the Han River.

Researches on climate change mostly belong to the type of "If–then–what". According to the output results of GCMs, specific scenario is still the basic way to help determine the possible impacts of future climate change, although there are some uncertainties^39^. The uncertainty of climate change impact assessment includes future emission scenarios, GCMs, downscaling techniques, hydrological models, etc. Many scholars have found that the selection of GCM is the biggest factor leading to the uncertainty of climate change impact assessment compared with other factors^38,40^. Figure 10 shows the uncertainty range of future runoff projection at four hydrological stations in the Han River. It is observed that runoff relative changes of GCM ensemble median output at Baihe, Ankang, Danjiangkou and Huangzhuang stations are all fluctuated up and down about zero. As for the 90% confidence interval, the relative changes at four stations are mainly within [− 50%, + 50%]. The simulation results are significant differences among different GCMs, which proves that GCM output is a main factor leading to the uncertainty. It is likely to draw biased conclusions if only one GCM is used to evaluate the impact of climate change on water resources, and the GCM ensemble median results can reduce the uncertainty of runoff projection.

Therefore, to reduce the uncertainty of future climate and runoff predictions, ten GCMs are selected in this study and historical meteorological data is used to test the historical scenario simulated by GCMs. The DBC method is used to correct the future precipitation and temperature in Han River basin, which can take into account both precipitation frequency and the distribution of precipitation. Through different GCMs and deviation correction method, the uncertainty of study can be reduced, and the rationality and practicability of hydrological simulation prediction results in Han River basin can be ensured.

## Conclusions

In this paper, the impacts of future climate change and future human activities on water availability and optimal allocation of water resources in the Han River basin are investigated. This work is conducted based on three levels: Firstly, the future climate and LUCC scenarios are generated and used to force the SWAT model to project future water availability. Secondly, the Quota method is used to predict water demand in the planning year. Finally, the optimal water resources allocation model is established to analyze the supply–demand balance results under scenarios, and then to quantify the impacts of future water availability and water demand on water resources allocation. The main conclusions are summarized below.

1. The annual water availability has an increasing tendency in the future impacted by future climate change and future human activities compared with the base period, while the growth rate of the whole basin in RCP4.5 is 4.47%.
2. In 2030, the total water demand of Han River basin at four annual average water flow frequencies (50%, 75%, 90%, and 95%) will be 35.18, 36.46, 37.84, and 38.33 billion m^3^, respectively, which shows a slight increase compared with the water demand in 2016.
3. A nonlinear relationship has been identified between the optimal allocation of water resources and the water availability while a linear association between the former and the water demand. The effects of future varied water supply combined future climate change and human activities (LUCC and water transfer projects) were assessed and implemented to alleviate water shortages of the case study, while the future varied water demand will increase water shortages of the case study.
4. To mitigate the negative impacts of the water donor area, especially on the downstream of the intake after water diversion in the upstream, the compensation mechanism should be established. The average water replenishment of the middle and lower reaches of the Han River should be 2.301 billion m^3^ in 2016 base year, and 4.495 billion m^3^ in 2030 planning year, respectively.

The research results of this paper can help to maintain the water resources management and ecological environment protection planning and design of the Han River basin under the co-change of climate and human activities in the future. In addition, the impacts of future climate change and human activities on runoff are different with different climatic characteristics and basin changes. Therefore, the developed framework in this study quantitatively shows the impacts of future changing environments on optimal allocation of water resources. The results may provide abundant information as references in guiding the allocation of water resources, and improves understandings of the assessments of water availability and water demand at a regional or national scale.

## Supplementary Information

Supplementary Information.
