# Supplementary material for: Adaptive optimal allocation of water resources response to future water availability and water demand in the Han River basin, China
Source: Sci Rep. 2021 Apr 12;11:7879. doi: 10.1038/s41598-021-86961-1 (PMC8041889; doi:10.1038/s41598-021-86961-1)
Supplement: Supplementary file 1 — Supplementary Information. [file 41598_2021_86961_MOESM1_ESM.pdf]

Supporting Information for

Adaptive optimal allocation of water resources response to future water availability and water demand in the Han River basin, China

Jing Tian[1], Shenglian Guo[1,*], Lele Deng[1], Jiabo Yin[1], Zhengke Pan[2], Shaokun He[1], Qianxun Li[1]

[1] State Key Laboratory of Water Resources and Hydropower Engineering Science, Wuhan University, Wuhan 430072, China

[2] Changjiang Institute of Survey, Planning, Design and Research, Wuhan, 430010, China

[*] Correspondence: slguo@whu.edu.cn

Table S1 Percentages of LUCC type area in the Han River basin (%)

Land use type	Historical 2010	Future 2030
Farmland	35.2	34.2
Forest land	40.0	41.4
Grassland	19.2	18.3
Water bodies	2.8	2.8
Construction land	2.7	3.2
Bare land	0.1	0.1

Table S2. The population in the 2016 base year and 2030 planning year of Han River basin.

Region	2016 base year ($\times10^4$)			2030 planning year ($\times10^4$)		
	Urban	Rural	Sum	Urban	Rural	Sum
U1	2.65	4.49	7.14	4.10	3.44	7.54
U2	137.86	217.23	355.10	219.59	157.04	376.63
U3	70.41	141.28	211.69	121.80	104.13	225.92
U4	111.18	209.40	320.59	182.82	156.54	339.36
U5	0.02	0.61	0.64	0.03	0.65	0.67
U6	2.55	4.73	7.27	3.68	3.95	7.63
U7	122.50	11.53	134.03	140.00	8.63	148.63
U8	108.65	213.78	322.44	153.97	197.86	351.84
U9	0.62	3.11	3.73	0.93	2.80	3.73
U10	44.12	67.05	111.18	67.08	52.73	119.81
U11	1.32	9.51	10.84	2.02	8.82	10.84
U12	58.67	101.91	160.58	79.31	97.83	177.14
U13	0.00	1.45	1.45	0.00	1.58	1.58
U14	2.91	2.45	5.36	3.80	2.12	5.92
U15	42.26	17.63	59.89	48.81	16.54	65.35
U16	0.63	4.75	5.38	0.97	4.42	5.38
U17	244.78	203.34	448.11	309.72	183.95	493.66

U18	361.80	486.90	848.70	521.70	381.63	903.33
U19	15.41	40.41	55.82	23.09	35.40	58.49
U20	1.07	4.37	5.44	1.45	4.47	5.92
U21	135.46	118.88	254.34	172.28	104.31	276.59
U22	57.93	120.97	178.90	77.09	115.35	192.43
U23	90.25	110.80	201.06	124.20	94.87	219.07
U24	19.55	20.30	39.85	25.59	16.66	42.25
U25	63.11	98.31	161.42	80.27	92.33	172.60
U26	119.39	10.03	129.42	135.21	8.27	143.48
Sum	1815.11	2225.22	4040.34	2499.50	1856.31	4355.81

Table S3. Water quota of domestic in the 2016 base year and 2030 planning year of Han River basin (L/day).

Region	2016 base year		2030 planning year	
	Urban	Rural	Urban	Rural
U1	99.75	58.92	123.50	70.97
U2	112.25	63.34	131.92	74.87
U3	97.52	60.15	123.04	70.80
U4	98.92	59.43	131.38	73.64
U5	122.80	55.00	142.50	70.00
U6	101.58	85.00	114.00	100.00
U7	116.38	46.50	133.00	55.00
U8	144.70	65.60	156.89	77.06
U9	80.75	70.00	95.00	85.00
U10	102.81	75.00	116.79	90.00
U11	80.75	70.00	95.00	85.00
U12	141.22	85.00	159.99	100.00
U13	0.00	77.17	0.00	90.78
U14	140.23	73.38	159.38	86.33
U15	133.41	75.79	151.04	89.17
U16	80.75	70.00	95.00	85.00
U17	145.70	85.78	164.99	100.9
U18	108.06	75.00	121.07	90.00
U19	93.61	70.00	107.69	85.00
U20	117.67	78.46	133.59	92.31
U21	143.18	91.07	162.11	106.97
U22	160.03	98.08	181.69	115.38
U23	136.97	89.55	155.49	105.18
U24	160.04	98.08	181.69	115.38
U25	160.02	98.08	181.69	115.38
U26	159.84	98.08	181.76	114.58

Table S4. Water quota of industrial user sector in the 2016 base year and 2030 planning year of Han River basin (L/day).

Region	2016 base year		2030 planning year	
	High water consumption industry	General industry	High water consumption industry	General industry
U1	177	65	64	35
U2	162	65	64	35
U3	277	112	93	29
U4	198	53	53	27
U5	55	41	20	15
U6	432	110	70	19
U7	0	84	0	35
U8	306	67	130	25
U9	62	39	26	16
U10	55	38	20	15
U11	65	43	25	19
U12	0	59	0	22
U13	421	96	179	36
U14	427	114	181	43
U15	422	96	179	36
U16	63	43	25	18
U17	422	93	179	35
U18	72	47	30	20
U19	410	86	174	32
U20	55	41	20	15
U21	361	80	153	30
U22	429	92	182	35
U23	374	88	159	33
U24	429	119	182	45
U25	390	86	166	33
U26	218	48	93	18

Note: The water quota of high water consumption industry in U7 and U12 is 0, which means that there are no high water consumption industries in these two regions.

Table S5. Water quota of agricultural user sector in the 2016 base year and 2030 planning year of Han River basin (m^3/a).

Region	2016 base year				2030 planning year			
	P=50%	P=75%	P=90%	P=95%	P=50%	P=75%	P=90%	P=95%
U1	101	117	133	133	99	115	131	131
U2	252	271	296	296	247	266	290	290
U3	237	254	276	276	232	249	271	271
U4	226	245	268	268	222	240	263	263
U5	153	83	103	110	150	81	101	107

U6	148	158	172	181	144	154	167	175
U7	82	100	113	129	77	95	109	121
U8	201	225	261	274	204	228	256	267
U9	203	225	256	256	218	242	273	273
U10	105	119	142	142	100	116	138	138
U11	200	227	253	253	216	246	271	271
U12	225	269	299	309	214	259	291	308
U13	230	293	382	408	230	293	382	408
U14	191	244	324	348	177	226	288	309
U15	202	233	281	326	209	237	283	334
U16	199	223	251	251	217	240	267	267
U17	210	254	287	321	200	225	257	280
U18	204	246	272	272	217	263	289	289
U19	205	240	292	292	219	256	311	311
U20	282	361	453	496	274	325	382	381
U21	250	269	297	312	241	251	269	282
U22	260	309	351	372	234	277	317	331
U23	202	230	264	292	193	215	242	269
U24	208	236	292	336	198	221	279	318
U25	194	232	271	297	188	218	256	279
U26	249	288	341	357	245	285	333	349

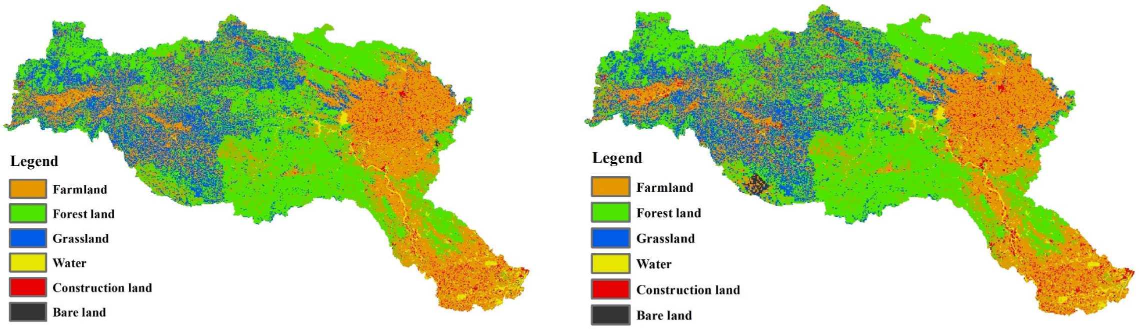

(a) LUCC2010 (b) LUCC2030

Fig.S1. LUCC scenarios of Han River basin. (This figure is generated by ArcGIS10.2 software. URL link: http://www.arcgisonline.cn/).

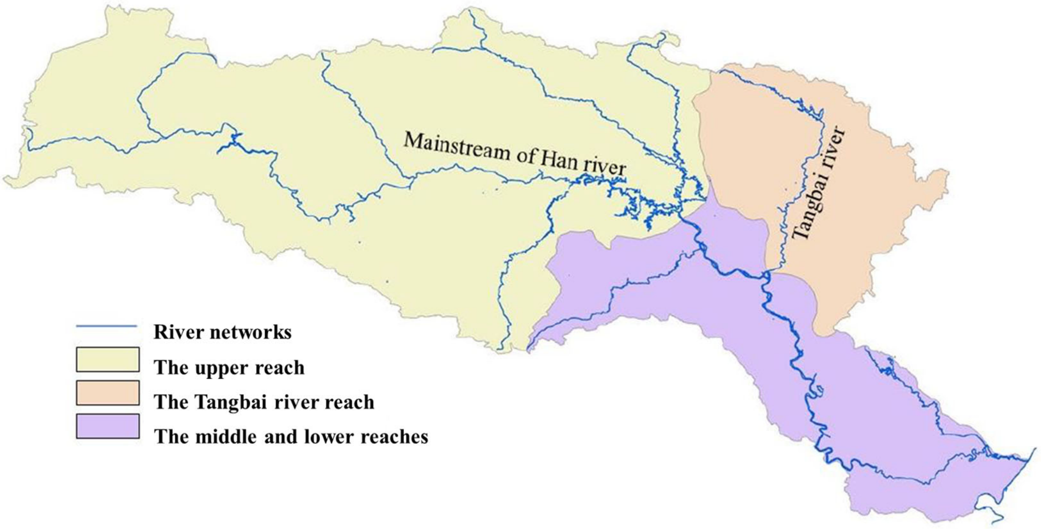

Figure S2. The upper, Tangbai river, middle and lower reaches of Han River basin. (This figure is generated by ArcGIS10.2 software. URL link: http://www.arcgisonline.cn/).
